# Supplementary figures and images for: Evidence of the Beneficial Impact of Three Probiotic-Based Food Supplements on the Composition and Metabolic Activity of the Intestinal Microbiota in Healthy Individuals: An Ex Vivo Study
Source: Nutrients. 2023 Dec 12;15(24):5077. doi: 10.3390/nu15245077 (PMC10745619; doi:10.3390/nu15245077)

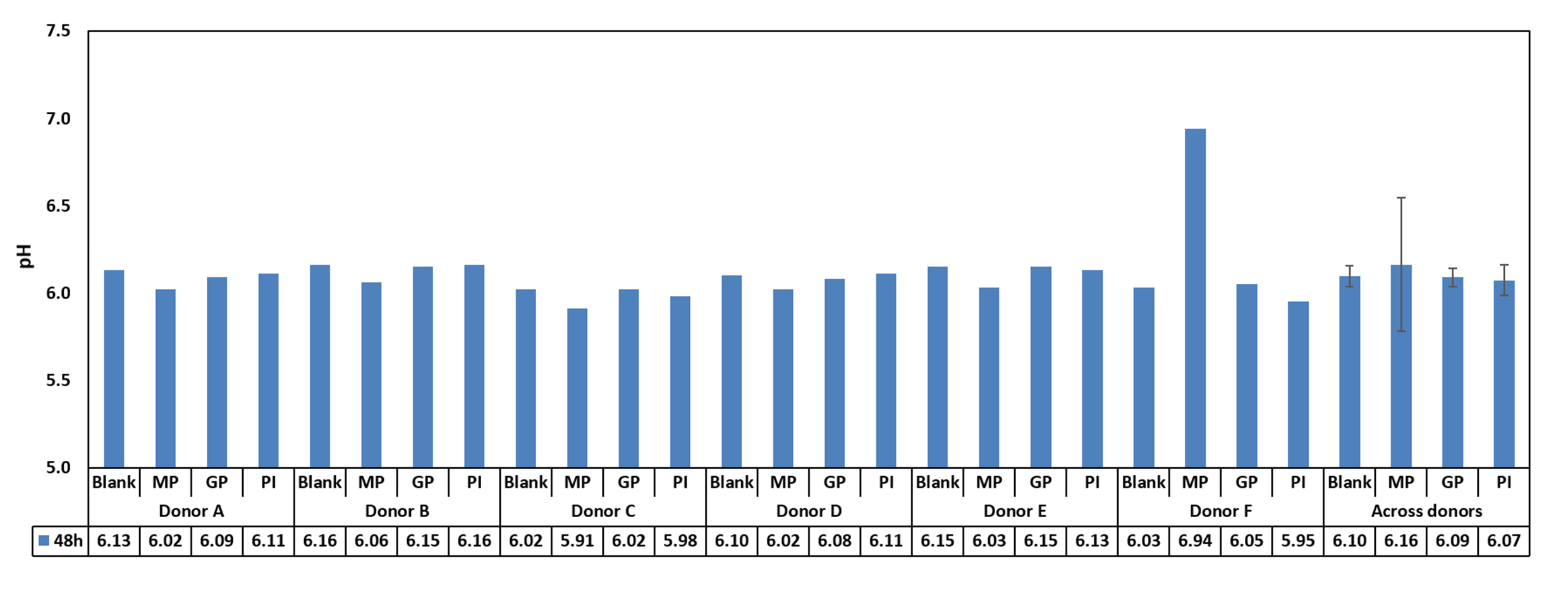

Supplement: Supplementary file 1 [file nutrients-15-05077-s001.zip › S-Figure 1.tif]

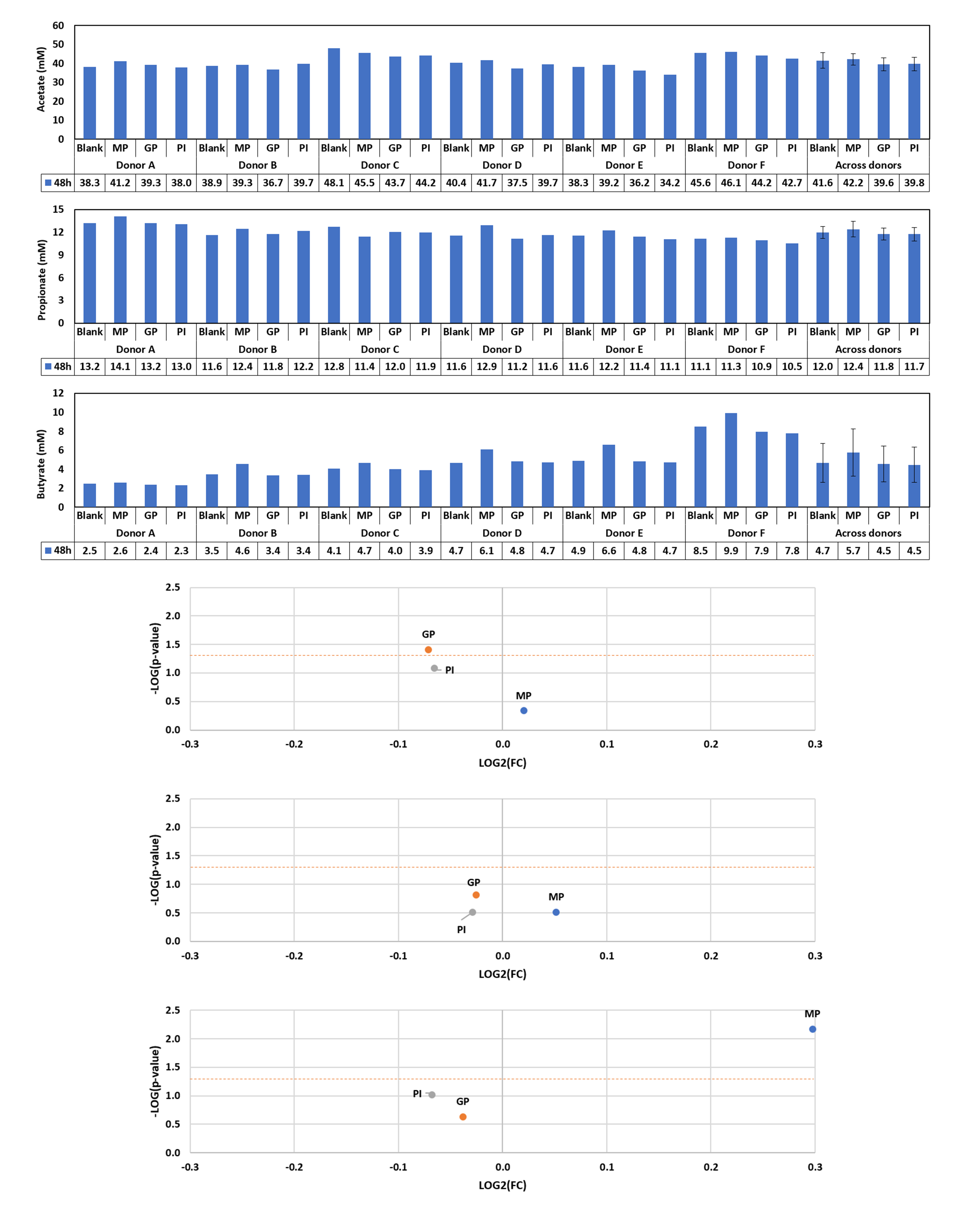

Supplement: Supplementary file 1 [file nutrients-15-05077-s001.zip › S-Figure 2.tif]

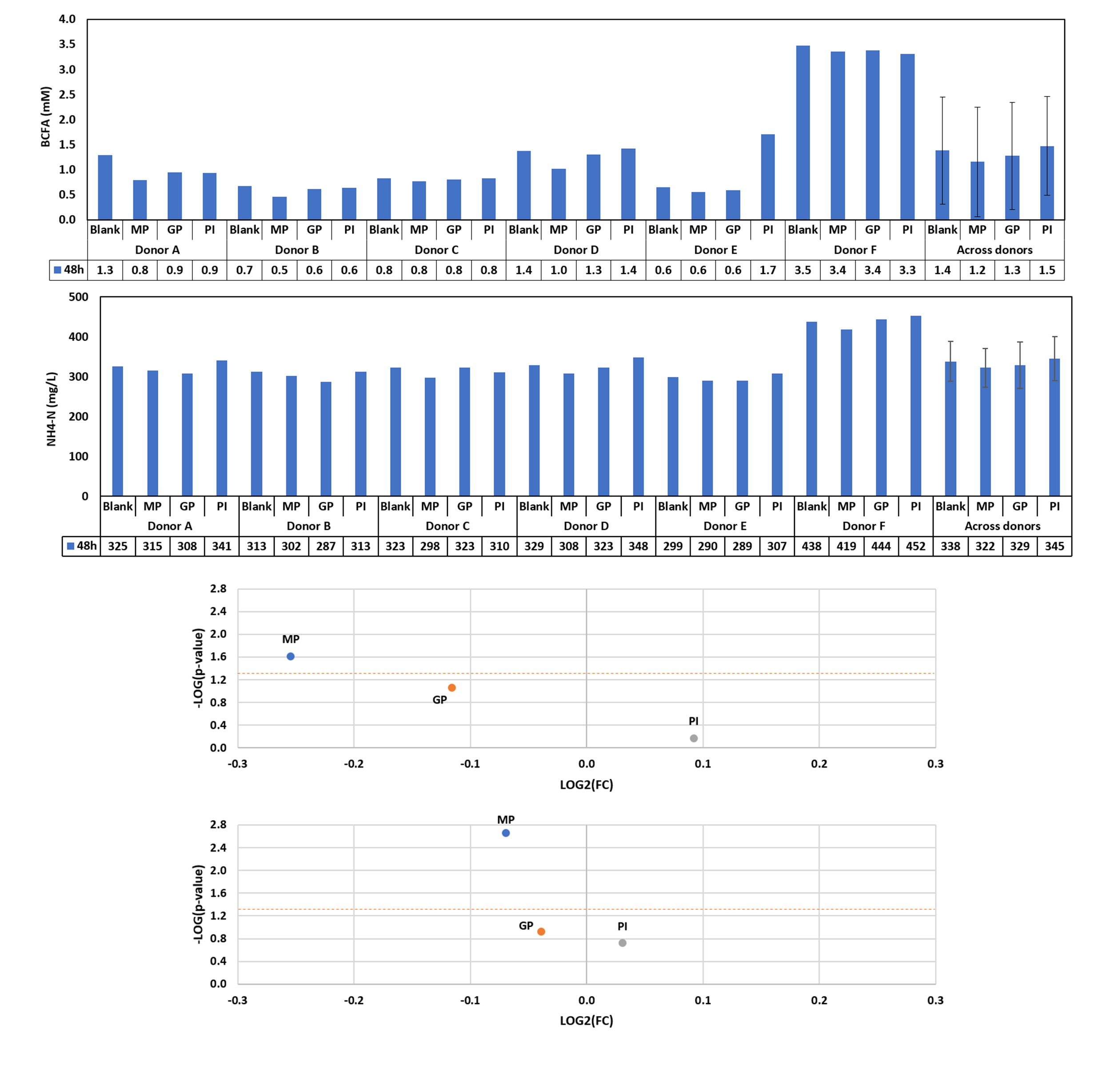

Supplement: Supplementary file 1 [file nutrients-15-05077-s001.zip › S-Figure 3.tif]

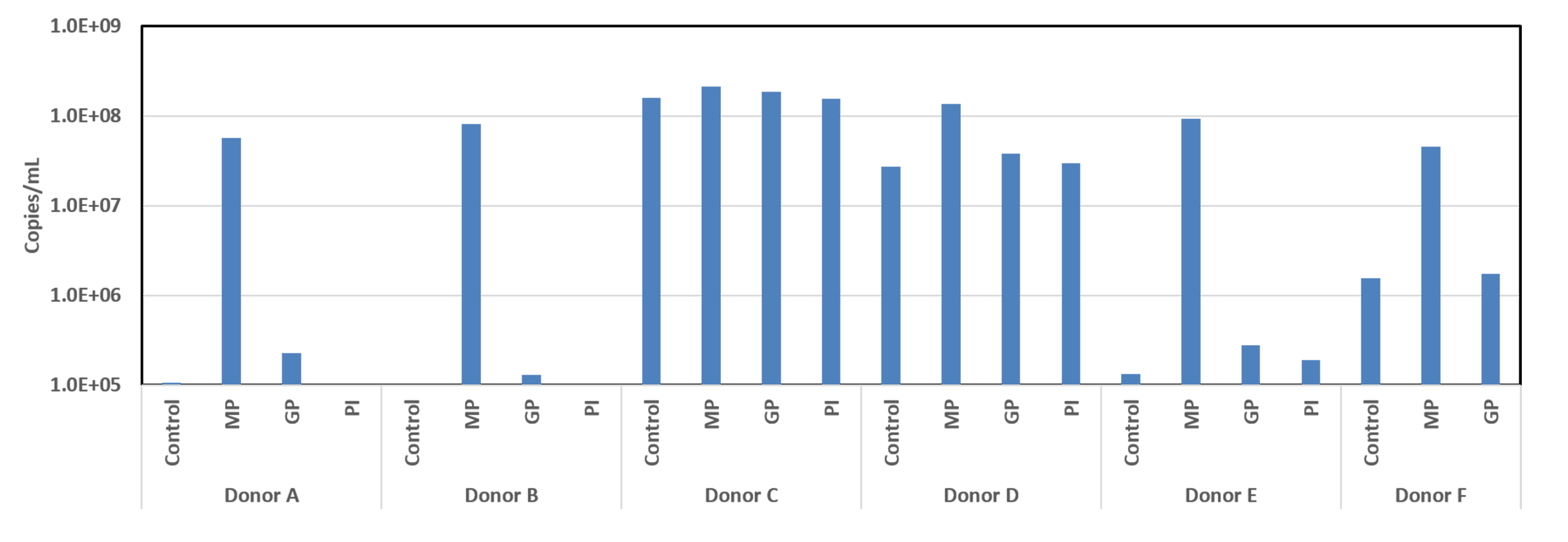

Supplement: Supplementary file 1 [file nutrients-15-05077-s001.zip › S-Figure 4.tif]

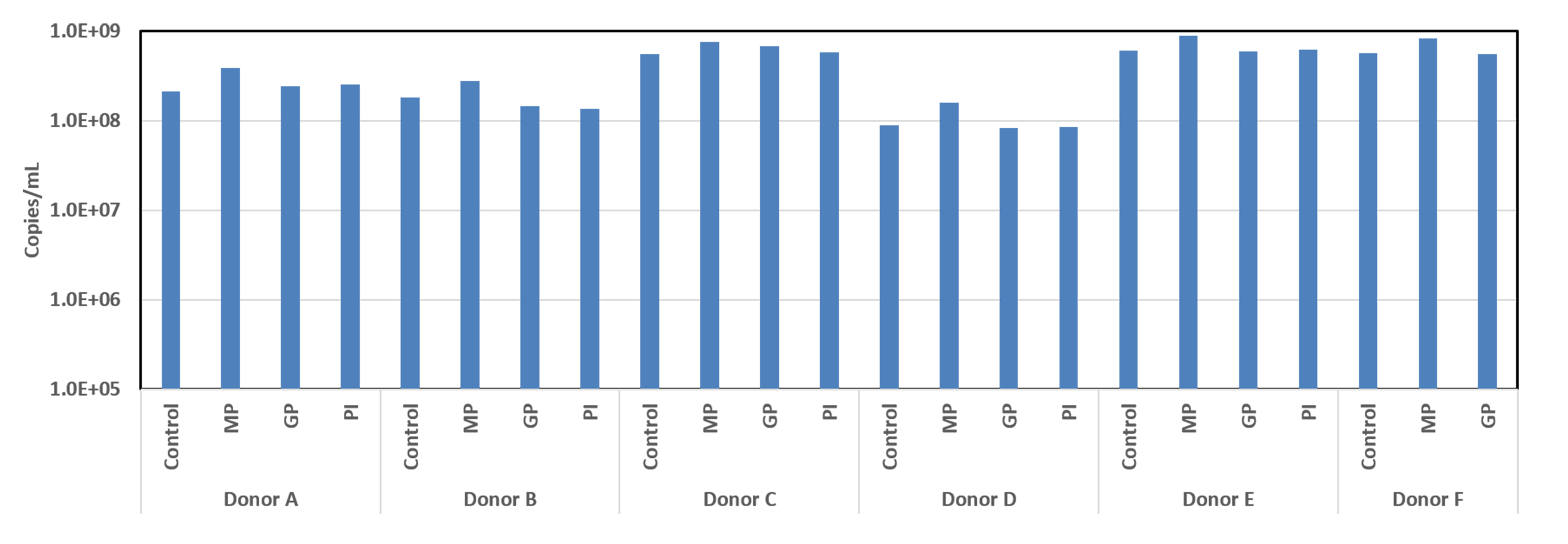

Supplement: Supplementary file 1 [file nutrients-15-05077-s001.zip › S-Figure 5.tif]

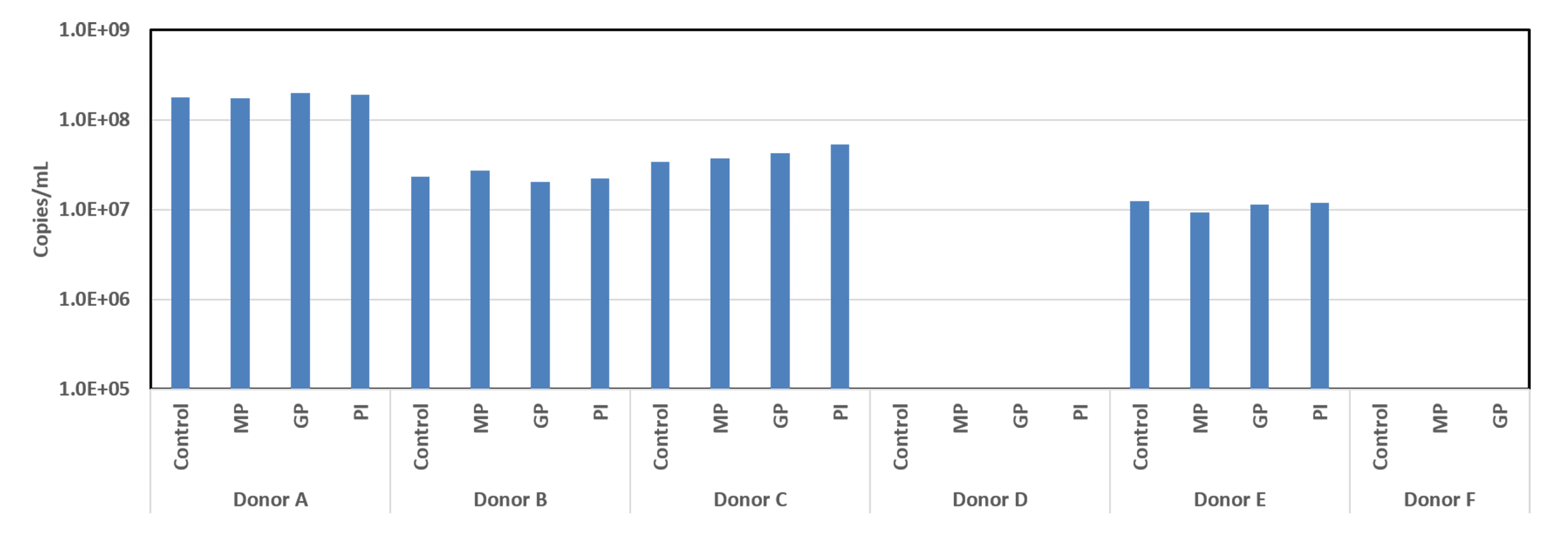

Supplement: Supplementary file 1 [file nutrients-15-05077-s001.zip › S-Figure 6.tif]

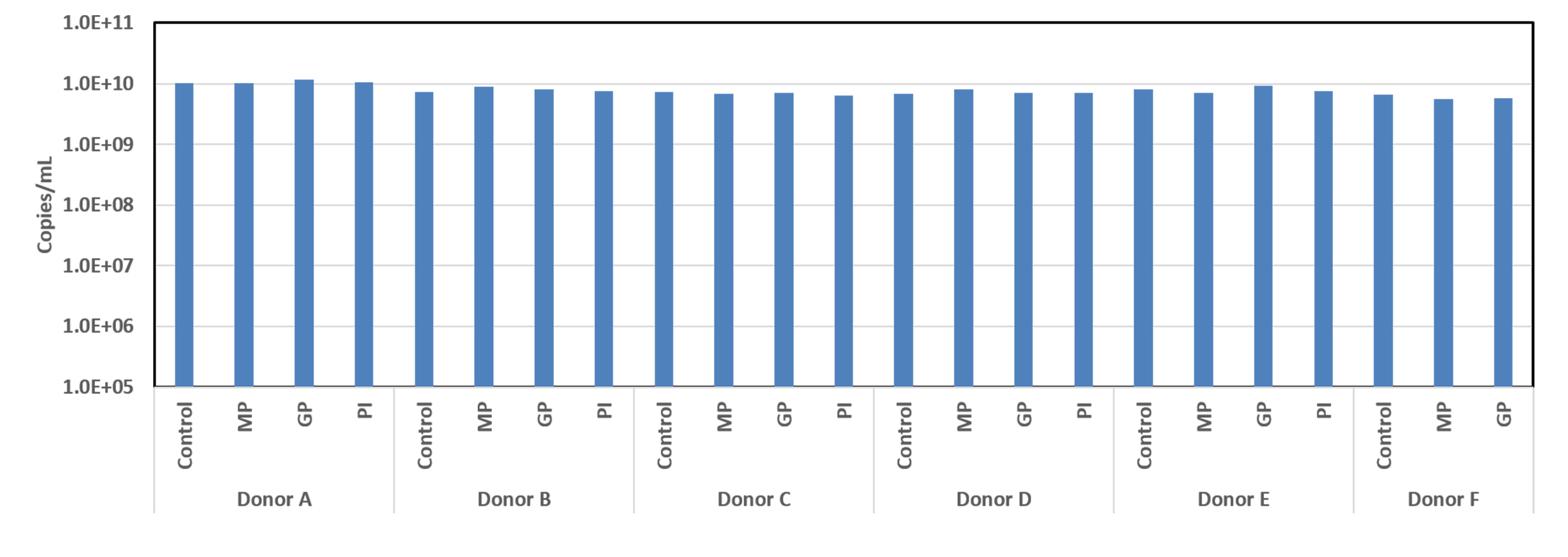

Supplement: Supplementary file 1 [file nutrients-15-05077-s001.zip › S-Figure 7.tif]

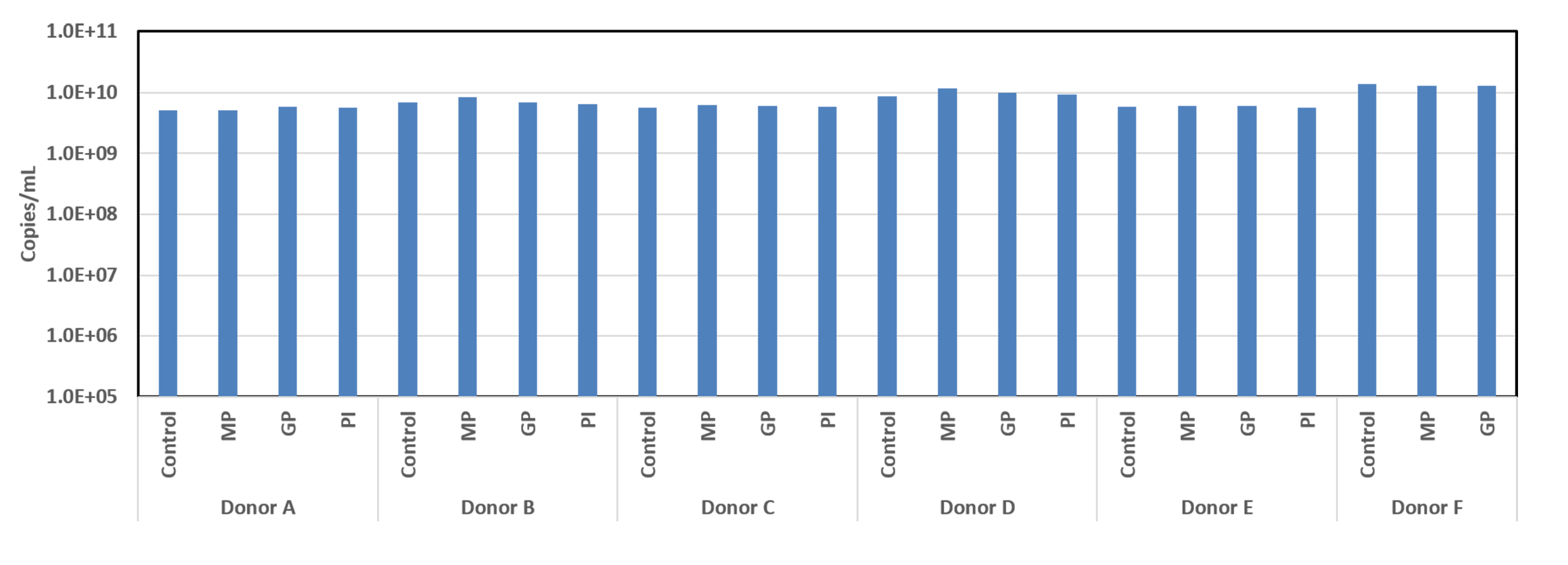

Supplement: Supplementary file 1 [file nutrients-15-05077-s001.zip › S-Figure 8.tif]
